# Development and evaluation of the Capability, Opportunity, and Motivation to deliver Physical Activity in School Scale (COM-PASS)

**DOI:** 10.1186/s12966-024-01640-4

**Published:** 2024-08-26

**Authors:** A. Verdonschot, M. R. Beauchamp, T. A. Brusseau, M. J. M. Chinapaw, L. B. Christiansen, A. Daly-Smith, N. Eather, S. J. Fairclough, G. Faulkner, L. Foweather, A. García-Hermoso, A. S. Ha, N. Harris, T. Jaakkola, R. Jago, S. G. Kennedy, N. J. Lander, C. Lonsdale, Y. Manios, E. Mazzoli, E. Murtagh, N. Nathan, P. J. Naylor, M. Noetel, B. O’Keeffe, G. K. Resaland, N. D. Ridgers, K. Ridley, N. Riley, R. R. Rosenkranz, S. K. Rosenkranz, A. Sääkslahti, S. M. Sczygiol, T. Skovgaard, E. M. F. van Sluijs, J. J. Smith, M. Smith, G. Stratton, J. Vidal-Conti, C. A. Webster, E. S. Young, D. R. Lubans

**Affiliations:** 1 Centre for Active Living and Learning, School of Education, University of Newcastle, Newcastle, Australia; 2 Active Living Research Program, Hunter Medical Research Institute, New Lambton Heights, New South Wales, Australia; 3 School of Kinesiology, University of British Columbia, Vancouver, British Columbia Canada; 4 Department of Health and Kinesiology, University of Utah, Salt Lake City, USA; 5 Department of Public and Occupational Health, Amsterdam UMC, Location Vrije Universiteit Amsterdam, Amsterdam Public Health Research Institute, Amsterdam, The Netherlands; 6 Active Living, Department of Sports Science and Clinical Biomechanics, University of Southern Denmark, Odense M, Denmark; 7 Faculty of Health Studies, University of Bradford, Bradford, UK; 8 Centre for Applied Education Research, Wolfson Centre for Applied Health Research, Bradford, UK; 9 Sport, Physical Activity, Health, & Wellbeing Research Group, and Department of Sport & Physical Activity, Edge Hill University, Ormskirk, UK; 10 Physical Activity Exchange, Research Institute of Sport and Exercise Sciences, Liverpool John Moores University, Liverpool, UK; 11 Navarrabiomed, Hospital Universitario de Navarra, Universidad Pública de Navarra, IdiSNA, Pamplona, Navarra Spain; 12 Department of Sports Science and Physical Education, Faculty of Education, The Chinese University of Hong Kong, Hong Kong SAR, China; 13 Human Potential Centre, Auckland University of Technology, Auckland, New Zealand; 14 Faculty of Sport and Health Sciences, University of Jyväskylä, Jyväskylä, Finland; 15 Population Health Sciences, Bristol Medical School, University of Bristol, Bristol, UK; 16 School of Health Sciences, Western Sydney University, Kingswood, New South Wales Australia; 17 Institute for Physical Activity and Nutrition (IPAN), School of Exercise and Nutrition Sciences, Faculty of Health, Deakin University, Geelong, Victoria Australia; 18 Institute for Positive Psychology and Education, Australian Catholic University, North Sydney, New South Wales Australia; 19 Department of Nutrition and Dietetics, School of Health Science and Education, Harokopio University, Athens, Greece; 20 Institute of Agri-Food and Life Sciences, University Research & Innovation Center, H.M.U.R.I.C., Hellenic Mediterranean University, Crete, Greece; 21 School of Health and Social Development, Faculty of Health, Deakin University, Geelong, Victoria Australia; 22 Department of Physical Education and Sport Sciences, University of Limerick, Limerick, Ireland; 23 Hunter New England Population Health, Hunter New England Local Health District, Wallsend, New South Wales Australia; 24 College of Health, Medicine and Wellbeing, School of Medicine and Public Health, The University of Newcastle, Newcastle, New South Wales Australia; 25 National Centre of Implementation Science (NCOIS), The University of Newcastle, Newcastle, New South Wales Australia; 26 Hunter Medical Research Institute, New Lambton Heights, New South Wales, Australia; 27 School of Exercise Science, Physical and Health Education, University of Victoria, Victoria, British Columbia Canada; 28 School of Psychology, The University of Queensland, Brisbane, Queensland Australia; 29 Centre for Physically Active Learning, Faculty of Education, Arts and Sports, Western Norway University of Applied Sciences, Sogndal, Norway; 30 Alliance for Exercise, Nutrition and Activity (ARENA), Allied Health and Human Performance, University of South Australia, Adelaide, South Australia Australia; 31 College of Education, Psychology and Social Work, Flinders University, Adelaide, South Australia Australia; 32 Department of Kinesiology and Nutrition Sciences, University of Nevada, Las Vegas, Las Vegas, Nevada USA; 33 Department of Neuromotor Behaviour and Exercise, University of Münster, Münster, Germany; 34 MRC Epidemiology Unit, University of Cambridge, Cambridge, UK; 35 School of Nursing, University of Auckland, Auckland, New Zealand; 36 Applied Sport Technology Exercise and Medicine Research Centre, Faculty Science and Engineering, Swansea University, Wales, UK; 37 Sport and Exercise Sciences, University of Western Australia, Perth, Western Australia Australia; 38 Physical Activity and Sport Sciences Research Group (GICAFE), University of the Balearic Islands, Palma, Spain; 39 Department of Kinesiology, Texas A and M University – Corpus Christi, Corpus Christi, Texas USA

**Keywords:** Implementation, Physical activity, Scale, Primary and secondary schools

## Abstract

**Background:**

Teachers are recognized as ‘key agents’ for the delivery of physical activity programs and policies in schools. The aim of our study was to develop and evaluate a tool to assess teachers’ capability, opportunity, and motivation to deliver school-based physical activity interventions.

**Methods:**

The development and evaluation of the Capability, Opportunity, and Motivation to deliver Physical Activity in School Scale (COM-PASS) involved three phases. In Phase 1, we invited academic experts to participate in a Delphi study to rate, provide recommendations, and achieve consensus on questionnaire items that were based on the Capability, Opportunity, and Motivation Behavior (COM-B) model. Each item was ranked on the degree to which it matched the content of the COM-B model, using a 5-point scale ranging from ‘1 = *Poor match’* to ‘5 = *Excellent match’*. In Phase 2, we interviewed primary and secondary school teachers using a ‘think-aloud’ approach to assess their understanding of the items. In Phase 3, teachers (*n* = 196) completed the COM-PASS to assess structural validity using confirmatory factor analysis (CFA).

**Results:**

Thirty-eight academic experts from 14 countries completed three rounds of the Delphi study. In the first round, items had an average rating score of 4.04, in the second round 4.51, and in the third (final) round 4.78. The final tool included 14 items, which related to the six constructs of the COM-B model: physical capability, psychological capability, physical opportunity, social opportunity, reflective motivation, and automatic motivation. In Phase 2, ten teachers shared their interpretation of COM-PASS via a 20-min interview, which resulted in minor changes. In Phase 3, CFA of the 3-factor model (i.e., capability, opportunity, and motivation) revealed an adequate fit to the data (χ^2^ = 122.6, *p* < .001, CFI = .945, TLI = .924, RMSEA = .066). The internal consistencies of the three subscale scores were acceptable (i.e., capability: α = .75, opportunity: α = .75, motivation: α = .81).

**Conclusion:**

COM-PASS is a valid and reliable tool for assessing teachers’ capability, opportunity, and motivation to deliver physical activity interventions in schools. Further studies examining additional psychometric properties of the COM-PASS are warranted.

**Supplementary Information:**

The online version contains supplementary material available at 10.1186/s12966-024-01640-4.

## Background

Regular participation in physical activity is essential for young people’s physical, psychological, emotional, and cognitive health [1]. However, only 27% to 33% of children and adolescents meet the recommended 60 min of moderate to vigorous physical activity per day across the globe [2]. Physical activity begins to decline during childhood and continues throughout adolescence [3, 4]. Although some of the decline in physical activity may have a biological basis, increased academic and work commitments (i.e., lack of time), low perceived competence, and lack of interest and support from peers have been identified as barriers to participation among adolescents [5, 6]*.* Schools are internationally recognized as key settings for promoting physical activity, given many children and adolescents attend school for a substantial portion of their time [7]. In addition, most education systems have policies and curricula that mandate physical activity opportunities for young people during school hours. Schools also have qualified personnel (i.e., teachers and support staff) responsible for supporting the education, health, and well-being of young people.

Despite their potential, school-based physical activity interventions have had limited effect on young people’s objectively measured physical activity [8–13]. For example, a recent individual participant pooled meta-analysis of randomized controlled trials found that school-based interventions led to increases of 1.5 min/day of vigorous-intensity and 1.3 min/day of moderate-intensity physical activity [12]. Jago and colleagues recently suggested that the failure to consider important school contextual factors (e.g., school setting, ethos, staff, and sociodemographic factors) has contributed to the small effects [14, 15]. Poor implementation of physical activity programs and policies by teachers and other school staff has been offered as another reason for the limited effects [16].

Teachers (i.e., generalist and specialist physical education) are recognized as ‘key agents of change’ responsible for the implementation of school-based physical activity interventions [17–19]. Considering their frontline position in implementing physical activity programs and policies in primary and secondary school settings with a range of related tasks (e.g., designing physical activity curricula, organizing sports activities, or coordinating active breaks during class time), there is an urgent need to consider the barriers and facilitators teachers experience in the delivery of interventions. Naylor and colleagues conducted a systematic review of the factors influencing the implementation of school-based physical activity interventions and found that ‘time’ was the most commonly cited barrier [20]. Other influencing factors were resource availability and quality (e.g., activity resources, personnel, facilities), and supportive school climate (e.g., shared vision and administrative support) [20]. Using the Theoretical Domains Framework as a guide, Nathan and colleagues also reviewed the barriers and facilitators that influence the implementation of physical activity policies in schools. Their review of 17 studies found the most commonly reported domains were 'environmental context and resources' (e.g., availability of equipment, time or staff), 'social influences' (e.g., support from school executives), ‘goals’(e.g., perceived priority of the physical activity policy) and 'skills' (e.g., teachers' capability to implement the policy) [21]. In summary, the most commonly reported barriers to the implementation of physical activity programs and policies in schools include inadequate teacher training, time constraints, lack of motivation, and low perceived priority. Failure to consider these factors (i.e., determinants of implementation) in the co-creation and feasibility stages, may help explain the modest effects of previous school-based interventions.

Given the multiple challenges experienced by teachers, there is a need to identify and evaluate the impact of school-based implementation support strategies (i.e., methods used to enhance the adoption and implementation of interventions) [22, 23]. Previous reviews have examined the effect of staff professional development within school-based physical activity interventions [24] and the specific features associated with intervention fidelity and student physical activity [25]. Lander and colleagues [24] found that teacher professional development sessions lasting one day or more, delivered using multiple formats, and including subject and pedagogical content were more effective. More recently, Ryan and colleagues [25] demonstrated the use of behavior change techniques, informed by the COM-B model, such as ‘Action planning’ and ‘Feedback on the behavior’, were associated with better implementation and increases in children’s physical activity.

Although previous studies have attempted to examine the impact of implementation strategies on the key determinants of teachers’ implementation of physical activity, most have relied on unvalidated tools (i.e., designed specifically for their study) [20, 26]. There are more than 60 implementation theories, models, and frameworks [27, 28]. We selected the Capability, Opportunity, and Motivation Behavior (COM-B) model for this study because it offers a robust framework for understanding behavior and has proven utility in guiding interventions [17, 29]. Moreover, the COM-B model is now included in the ‘Individuals’ domain of the updated ‘Consolidated Framework for Implementation Research (CFIR)’, which is one of the most highly cited frameworks in implementation science [30]. Utilizing the COM-B model to assess teachers' capability, opportunity, and motivation to implement physical activity interventions within schools may offer insights into teacher-level determinants of implementation, and the way in which these may impact implementation of interventions. Such insights are essential for informing the development and evaluation of teacher delivered physical activity interventions. Therefore, the aim of our study was to develop and evaluate a brief tool for assessing teachers' capability, opportunity, and motivation to implement physical activity programs and policies in schools. The tool was designed to be adaptable, making it appropriate for the evaluation of different physical activity programs and policies in primary and secondary school settings.

## Methods

**Fig. 1:**
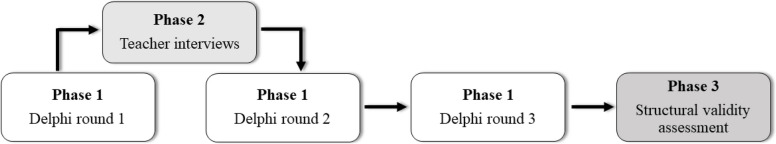
Phases of the development of the COM-PASS

Our study involved three research phases (see Fig. 1). In Phase 1, we explored items for the Capability, Opportunity, and Motivation to deliver Physical Activity in School Scale (COM-PASS) through a Delphi study with academic experts. In Phase 2, we assessed how teachers interpreted the COM-PASS items, and refined the tool using 'think-aloud' interviews with primary and secondary school teachers. In Phase 3, we explored the structural validity of the COM-PASS scores using confirmatory factor analyses (CFA) and structural equation modelling. The COM-PASS was designed to assess teachers’ capability, opportunity, and motivation to deliver specific physical activity interventions (i.e., programs or policies). The measure was not designed to assess teachers’ general capability, opportunity, and motivation to promote physical activity in school. Ethics approval was obtained from the University of Newcastle Human Research Ethics Committee (H-2021–0418) and the New South Wales Department of Education (State Education Research Application Process (SERAP): 2,022,215).

### Phase 1: Delphi study—scale development and content validity assessment

The aim of the first phase was to develop items for the COM-PASS and assess content validity using a Delphi study approach [31]. International academic experts (*n* = 45) who were first or senior author on a peer reviewed school-based physical activity intervention in the last five years were invited to review the COM-PASS tool by completing three review rounds of a 20 min (per round) online survey using the QuestionPro software [32]. The first version of the tool (round 1) included 13 items, and was based on items developed by Keyworth et al. [33] using the COM-B model [29] (see Supplementary File 1).

Two researchers (A.V. and D.R.L.) adapted the scale developed by Keyworth et al. [33] for physical activity promotion in the school setting. Academic experts were then asked to rank each item on the degree to which it matched the definition of the six COM-B model constructs: (i) *physical capability*, (ii) *psychological capability*, (iii) *physical opportunity,* (iv) *social opportunity,* (v) *reflective motivation* and (vi) *automatic motivation* [29] using a 5-point scale ranging from ‘1 = *Poor match*’ to’5 = *Excellent match*’. The survey included space for experts to make amendments and provide suggestions. The academic experts were informed their contribution would include three rounds including a 20-min online survey per round to provide their feedback. Academic experts who accepted the invitation were requested to complete their feedback within two weeks. A reminder was sent to the experts who did not complete the survey after the given time and extra time was given if requested. A.V. and D.R.L. reviewed the feedback per round and amended the questions accordingly, until the item rating reached an average score of 4.50 out of 5 or higher. The feedback was reviewed per round and amended accordingly. Prior Delphi studies have utilized cut-off thresholds ranging from 55 to 100% [31, 34]. However, in light of our COM-PASS items being grounded in the existing COM-B constructs, we used a consensus threshold of ≥ 4.50 out of a total of 5. The total timeframe of the Delphi study was eight months (November 2022 to July 2023).

### Phase 2: Teacher interviews—teachers’ interpretation assessment

In Phase 2, we recruited primary (*n* = 5) and secondary (*n* = 5) school teachers currently teaching in Australia via convenience sampling within our networks. The main aim of this phase was to evaluate how teachers understood and interpreted the COM-PASS items. Seeking input from members of the target population can offer valuable insights into both content relevance and representativeness [35, 36] and substantive aspects of validity [35]. We discussed the second version of the COM-PASS (i.e., after processing expert feedback on the first version) using a modified ‘think-aloud’ interview protocol [37–39] to further refine and pre-test the initial 17 items and response options including a 5-point Likert scale ranging from ‘1 = *Strongly disagree*’ to ‘5 = *Strongly agree*’.

Primary and secondary teachers completed an online (*n* = 8) or face-to-face 20-min interview (*n* = 2) with one author (A.V.). All interviews were audio and/or video recorded after obtaining consent. The teachers were instructed to read all COM-PASS items out loud and answer for all items separately the question ‘*What, in your own words, does the question mean to you?*’. Subsequently, the participants answered the following questions regarding the overall tool (a)*’Did the answer choices include your answer?*’, (b)*’Did you understand how to answer the questions?*’, (c) ‘*Did the questionnaire leave anything out you felt was important?*’ [37, 38] and (d)*’Do you have any other comments?*’. The interview script and the COM-PASS items used for this assessment can be found in Supplementary file 2. All interviews were transcribed (A.V.), reviewed (A.V. and D.R.L.) and amended accordingly (presented in Table 2 in the results section). We used a constant comparison approach [40] to identify sentences and phrases in which teachers raised concerns regarding one or more items, focusing on problematic and alternative interpretations of items. Participants received a 20-dollar (AUS) gift voucher to acknowledge their contribution. Detailed transcripts were attached to the email invitation for the academic experts as part of their second time reviewing the COM-PASS tool to evaluate to what extent the items matched to the COM-B constructs (Phase 1: Delphi study, round 2).

### Phase 3: Structural validity assessment

In Phase 3, we explored the structural validity of scores derived from the COM-PASS in a different sample of primary and secondary school teachers to Phase 2 [35, 41]. Participants were recruited using convenience sampling. First, we recruited teachers attending two Australian teacher physical education conferences (i.e., the Personal Development, Health and Physical Education Conference in New South Wales and the Australian Council for Health, Physical Education and Recreation Conference in Victoria). Second, we sent email invitations to our network of teachers in Australia, Germany, and the United Kingdom. Finally, we invited teachers from an ongoing implementation-effectiveness trial of the Australian *Resistance Training for Teens* program [42].

The COM-PASS items were included in a brief 10-min survey that included a 3-min video describing the *Resistance Training for Teens* (RT4T) program [42]. Teachers were asked to use RT4T as a reference when completing the COM-PASS items. We used CFA to explore structural validity because the COM-PASS tool was developed using the COM-B model [43]. We conducted analyses using IBM SPSS AMOS 29.0 software [44] and report the following fit indices: i) the comparative fit index (CFI) [45], ii) the Tucker-Lewis index (TLI) [46], and iii) the root mean square error of approximation (RMSEA) [47]. CFI and TLI compare the fit of a hypothesized model with the worst fit [48], while the RMSEA assesses how far a hypothesized model is from a perfect model. Hu and Bentler suggest that CFI and TLI values larger than 0.95 and an RMSEA value smaller than 0.06, indicate relatively good model fit to the observed data [45]. Our CFA included correlated residuals, as failing to correlate residuals may lead to parameter bias [49]. Additionally, Cronbach alphas were calculated to evaluate the measurement reliability of the separate capability, opportunity, and motivation constructs. Missing data were handled by the item mean substitution method where the mean item score was substituted for every missing value of a particular item, which has been identified as an appropriate approach if the number of items were missing for each scale are 20% or less [50]. The readability of the final tool was assessed using the Flesch Reading Ease Score to indicate its suitability for use with teachers, using a 100-point scale ranging from ‘0 = *Very difficult*’ to ‘100 = *Very easy*’ [51].

## Results

### Phase 1: Delphi study – scale development and content validity assessment

Three ranking review rounds were completed by 38 academic experts (84.4% response rate). The first round had an average score of 4.04, the second round 4.51, and the third (final) round had an average score of 4.78 agreement. This third round was deemed the final version, as all items received an average score of ≥ 4.50 (see Table 1). Although one item (Q14) scoring slightly below our chosen threshold at 4.45, we decided to retain the item after careful consideration of received comments.

**Table 1:** Results of the third ranking Delphi round of the COM-PASS by academic experts

COM-B constructs and COM-PASS items	Average score^b^
Physical capability Definition^a^: ‘Physical skill, strength or stamina’	
Q1: I have the physical fitness (e.g., aerobic and muscular fitness, flexibility) to deliver the [*physical activity program or policy*]	4.68
Q2: I have the physical skills (e.g., I can demonstrate the activities) to deliver the [*physical activity program or policy*]	4.84
Psychological capability Definition^a^: ‘Knowledge or psychological skills, strength, or stamina to engage in the necessary mental processes’	
Q3: I know how to deliver the [*physical activity program or policy*]	4.71
Q4: I can deliver the [*physical activity program or policy*] even when barriers emerge (e.g., lack of student engagement or lack of time)	4.55
Physical opportunity Definition^a^: ‘Opportunity afforded by the environment involving time, resources, locations, cues, physical ‘affordance’’	
Q5: My school has the physical facilities (e.g., access to a gym or appropriate indoor or outdoor space) to deliver the [*physical activity program or policy*]	4.89
Q6: My school has the equipment (e.g., resistance bands, balls, activity cards) to deliver the [*physical activity program or policy*]	4.87
Q7: I have enough time to plan the delivery of the [*physical activity program or policy*]	4.89
Social opportunity Definition^a^: ‘Opportunity afforded by interpersonal influences, social cues and cultural norms that influence the way that we think about things, e.g., the words and concepts that make up our language’	
Q8: I have the necessary support from school executives (e.g., principal or head of department) to deliver the [*physical activity program or policy*]	4.84
Q9: I have the necessary support from my colleagues to deliver the [*physical activity program or policy*]	4.89
Q10: I have the necessary support from parents and guardians to deliver the [*physical activity program or policy*]	4.84
Reflective motivation Definition^a^: ‘Reflective processes involving plans (self-conscious intentions) and evaluations (beliefs about what is good and bad)’	
Q11: I can see the benefits (e.g., improvements in students’ classroom behavior) of delivering the [*physical activity program or policy*]	4.82
Q12: I am motivated to deliver the [*physical activity program or policy*]	4.84
Automatic motivation Definition^a^: ‘Automatic processes involving emotional reactions, desires (wants and needs), impulses, inhibitions, drive states and reflex responses’	
Q13: I enjoy delivering the [*physical activity program or policy*]	4.71
Q14: Delivering the [*physical activity program or policy*] can become part of my school routine	4.45

^a^Michie, S., Atkins, L., & West, R. (2014). The behavior change wheel. *A guide to designing interventions. 1st ed. Great Britain: Silverback Publishing*, 1003–1010

^b^Using a 5-point scale ranging from ‘1 = *Poor match*’ to’5 = *Excellent match*’

### Phase 2: Teacher interviews—teachers’ interpretation assessment

We conducted interviews with primary (*n* = 5) and secondary (*n* = 5) school teachers (approximately 20 min in duration) to assess their interpretation of the COM-PASS. The second version of the COM-PASS (i.e., after review round 1 was completed by the experts) was used for this phase so any amendments could be approved by the academic experts in the following review round. Teachers’ interpretation was well aligned with the meaning of all COM-PASS items based on the COM-B model [29]. All teachers agreed on the question ‘*Did the answer choices include your answer?*’ and half of the teachers commented in their answer that the tool and answer options were clear and achievable to answer. The question ‘*Did you understand how to answer the questions?*’ was answered with ‘*yes*’ by all teachers. Regarding the question ‘*Did the questionnaire leave anything out you felt was important?*’, all teachers mentioned nothing was left out, except for one teacher who suggested to add in the question ‘*How easy did you find it to use the program materials/resources?*’ as they experienced challenges with a program application for tablets in their school and could not use it as much as they wanted due to technical issues. This item was added to the revised version of the COM-PASS (round 2) and reviewed by the academic experts to ensure the item was representative of the construct. However, this item was subsequently removed based on a low score of 4.03 and comments received from the academic experts (e.g., the item fits more in a process evaluation), and discussions among authors. Teachers had no further comments on the question ‘*Do you have any other comments?*’, and half of the teachers expressed appreciation for the tool and referred to the COM-PASS as a clear questionnaire.

As a result of three review rounds by the experts (Phase 1) and the ‘think-aloud’ interviews with teachers (Phase 2) the COM-PASS tool was refined three times whereby concerns from experts and teachers were discussed (A.V. and D.R.L.), resulting in actions taken (see Table 2). Changes to the final tool included: examples in five questions were amended to provide greater clarity, the addition of four items, three items were removed, two items were reworded, and two items were merged.

**Table 2:** Findings from the ‘think-aloud’ teacher interviews and expert reviewers

**Concerns** (items according to initial tool)	**Examples**	**Action taken** (items according to final tool)
On the terms ‘physical fitness’ (Q1) and ‘physical skills’ (Q2).	“Just wondering if everyone will understand physical fitness in the same way” [ER5].	Examples were added to both items (Q1 and Q2) to clarify ‘physical fitness (e.g., aerobic and muscular fitness, flexibility)’ and’physical skills (e.g., I can demonstrate the activities)’.
On the term ‘confidence’ (Q4).	“What is confidence here related to (self-confidence) or (program-confidence)—it might be a bit unclear (and a little hard for us to translate to Danish) I miss something like competence here—it could be specific didactical or pedagogical competences—or competences related to managing physical activity for kids.” [ER28].	Replaced the item on confidence with ‘I can deliver the [*physical activity program or policy*] even when barriers emerge (e.g., lack of student engagement or lack of time)’ (Q4).
On the use of ‘the time to prepare to deliver’ (Q7).	“The only question/observation I'd have corresponds to Q7 which seems to be more about the opportunity to prepare than opportunity to deliver.” [ER42].	Rephrased the wording into ‘I have enough time to plan the delivery of the [*physical activity program or policy*]’ (Q7).
On the excessive representation of authority figures and the lack of peers’ or parents’ perspective (Q8 and Q9).	“These have tapped into leadership support, but what about peer support? My colleagues consider the physical activity program to be worthy of delivery? (or see the value in the program)?’’ [ER44].	The two items on support from school executives and departmental support are merged into one and items on support from colleagues (Q9) and parents (Q10) are added.
On the use of ‘the benefits of delivering the [*physical activity program or policy]’* (Q10).	“ … what lays within benefits, is it their pupils, their physical activity levels or something else?” [ER19].	An example was added to clarify: ‘the benefits (e.g., improvements in students’ classroom behavior) of delivering the [*physical activity program or policy]’* (Q11).
On the categorization of ‘I am motivated to’ (Q11) as automatic motivation and on the measurement of the motivation of other teachers and students (Q12 and Q13).	“Q11 does not get at the automaticity but bringing it to mind like this is likely to tap the reflective portion. Q12 & Q13 seem to align with social opportunity. Maybe look more at some unconscious processes, habits, routines, etc. Regarding delivering one of the physical activity programs, it may be something that won't be there until they have been delivering the physical activity programs. The process of change for something like this would be reflective first, and then a shift toward automation.” [ER43].	Re-categorized item ‘I am motivated to deliver the [*physical activity program or policy*]’ under reflective motivation (Q12) and removed the items on other teachers’ and student’ motivation and added two items that reflects ones automatic motivation: ‘I enjoy delivering the [*physical activity program or policy*]’ (Q13) and ‘Delivering the [*physical activity program or policy*] can become part of my school routine’ (Q14).
On the item ‘I am planning to deliver the [*physical activity program of policy]’.* (Q13 from second version of the tool).	“I don’t know what that would mean. Are we already planning to deliver it?” [TI7].	Rephrased the item into: ‘Delivering the [*physical activity program or policy*] can become part of my school routine’ (Q14) to clarify.

*TI* Teacher interview, *ER* Expert reviewer

### Phase 3: Structural validity assessment

**Fig. 2:**
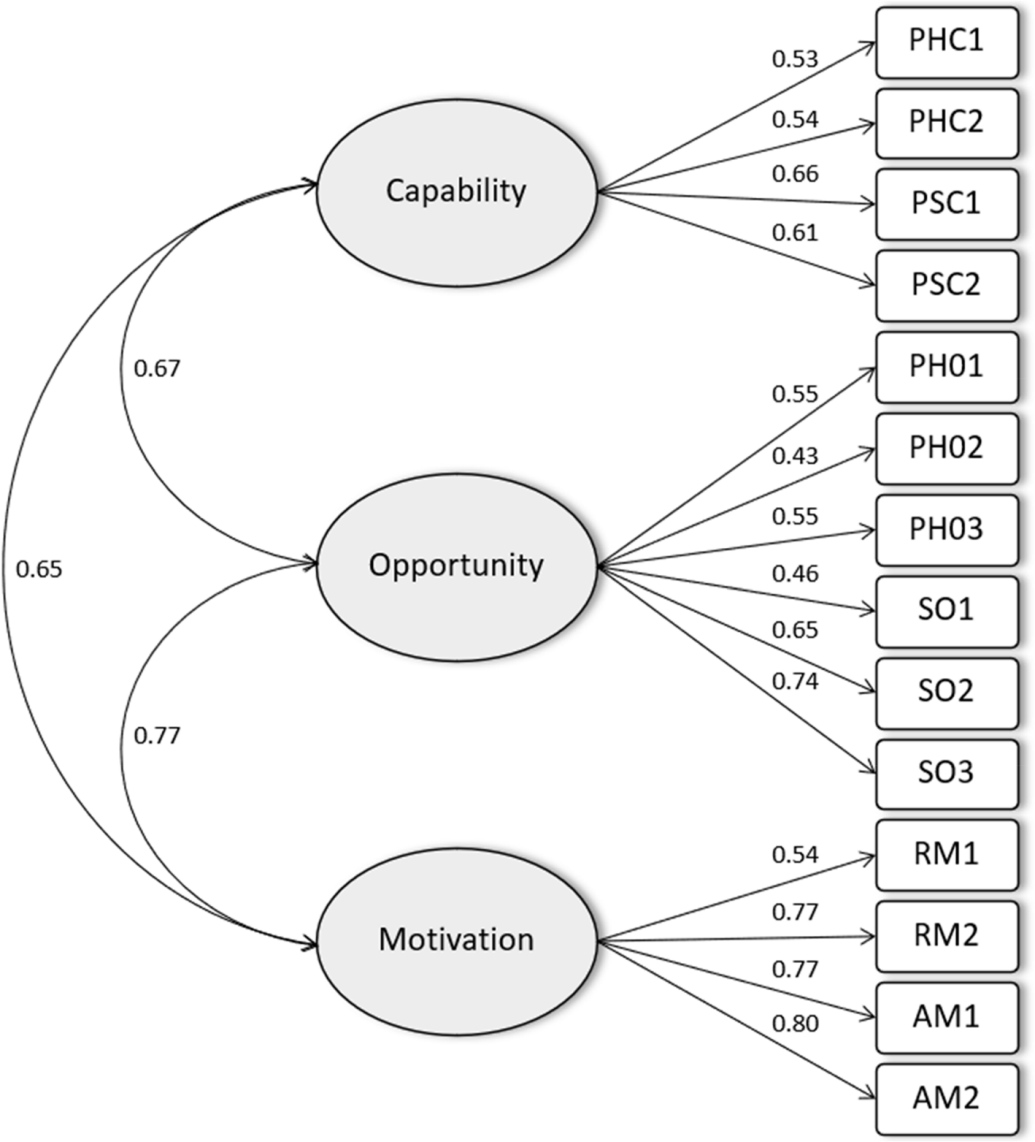
Standardized factor loadings and inter-factor correlations from the COM-PASS confirmatory factor analysis

In Phase 3, the final version of the COM-PASS was completed online by 196 teachers [male *n* = 100 (51%), female *n* = 96 (49%), primary *n* = 44 (22%) and secondary *n* = 152 (78%), Australian *n* = 155 (79%), German *n* = 10 (5%), and British *n* = 31 (16%)] (see Table 3). Teachers used the *Resistance Training for Teens* program as a reference when completing the scale [42]. Three missing values (0.1% of total responses) were replaced by the mean values of that specific item. Internal consistency was confirmed for all constructs (i.e., capability: α = 0.75, opportunity: α = 0.75, motivation α = 0.81). Supplementary file 3 presents the correlations among the COM-PASS items and the descriptive statistics (i.e., mean (*M),* standard deviation (*SD),* minimum, maximum and sample size). The final version of the COM-PASS obtained a Flesch Reading Ease Score of 54.6, equivalent to a reading level of 10th to 12th grade of high school [51]. Figure 2 presents an overview of the CFA using the IBM SPSS AMOS 29 Graphics software [44] with the three-factor loading model containing factors: capability, opportunity, and motivation. Findings from the CFA with the three components aligned with the COM-B model constructs (i.e., capability, opportunity, and motivation) demonstrating adequate fit (χ2 = 122.6, *df* = 66, *p* < 0.001, CFI = 0.945, TLI = 0.924, RMSEA = 0.066) and standardized factor loadings ranged from 0.43 to 0.80. A final version of the COM-PASS including answer options using a 5-point Likert scale anchored by 1 (Strongly disagree) to 5 (Strongly agree) can be found in Appendix 1.

**Table 3:** Internal consistency of the final of COM-PASS items and constructs

COM-B constructs	COM-PASS items	Cronbach Alpha (α)	
**Capability**			.75
Physical capability	PHC1	I have the physical fitness (e.g., aerobic and muscular fitness, flexibility) to deliver the *Resistance Training for Teens program*	
	PHC2	I have the physical skills (e.g., I can demonstrate the activities) to deliver the *Resistance Training for Teens program*	
Psychological capability	PSC1	I know how to deliver the *Resistance Training for Teens program*	
	PSC2	I can deliver the *Resistance Training for Teens program* even when barriers emerge (e.g., lack of student engagement or lack of time)	
**Opportunity**			.75
Physical opportunity	PHO1	My school has the physical facilities (e.g., access to a gym or appropriate indoor or outdoor space) to deliver the *Resistance Training for Teens program*	
	PHO2	My school has the equipment (e.g., resistance bands, balls, activity cards) to deliver the *Resistance Training for Teens program*	
	PHO3	I have enough time to plan the delivery of the *Resistance Training for Teens program*	
Social opportunity	SO1	I have the necessary support from school executives (e.g., principal or head of department) to deliver the *Resistance Training for Teens program*	
	SO2	I have the necessary support from my colleagues to deliver the *Resistance Training for Teens program*	
	SO3	I have the necessary support from parents and guardians to deliver the *Resistance Training for Teens program*	
**Motivation**			.81
Reflective motivation	RM1	I can see the benefits (e.g., improvements in students’ classroom behavior) of delivering the *Resistance Training for Teens program*	
	RM2	I am motivated to deliver the *Resistance Training for Teens program*	
Automatic motivation	AM1	I enjoy delivering the *Resistance Training for Teens program*	
	AM2	Delivering the *Resistance Training for Teens program* can become part of my school routine

## Discussion

The aim of our study was to develop and evaluate a brief tool for assessing teachers’ capability, opportunity, and motivation to deliver physical activity programs and policies in schools. Our findings provide preliminary support for the internal consistency and structural validity of scores derived from the COM-PASS in primary and secondary school teachers. The measure was designed to evaluate the effects of implementation support strategies in school-based physical activity interventions in efficacy, effectiveness, and dissemination studies. The COM-PASS may also have utility for evaluating the effects of pre-service (university undergraduate students) and in-service (current teachers) professional learning courses focused on physical activity promotion in schools.

It has been suggested that teacher professional development to support the delivery of school-based physical activity interventions should be informed by relevant theory and include evidence-based behavior change techniques [25]. However, prior to our study, we were not aware of any validated measures designed to assess teachers’ capability, opportunity, and motivation to deliver physical activity programs in schools. Importantly, our brief measure has been designed to be used to evaluate different physical activity programs and policies in research across the research translation pathway (i.e., from feasibility to dissemination). McKay and colleagues [28] recently proposed a minimum set of implementation outcomes (i.e., adoption, dose delivered, reach, fidelity, and sustainability) and determinants (i.e., context, acceptability, adaptability, feasibility, compatibility, cost, culture, dose, complexity, and self-efficacy) for the evaluation of physical activity interventions delivered at-scale. The COM-PASS overlaps with some of the determinants outlined by McKay and colleagues (e.g., self-efficacy), but is focused at the teacher level, as teachers are largely responsible for the delivery physical activity interventions in schools. In addition, the COM-PASS has been design for use in feasibility, efficacy, and effectiveness trials.

The COM-PASS has good content and structural validity and is considered appropriate by teachers. Positive feedback from teachers highlighted the user-friendly nature of the tool [52], which had a Flesch Reading Ease Score of 54.6 (i.e., reading level 10th to 12th grade of high school) [51]. All of the final items were scored ≥ 4.50 by academic experts, indicating that the COM-PASS items are well aligned with the COM-B model [29]. Findings from our CFA suggest that scores derived from the COM-PASS fit a three-factor model, aligned with the COM-B model (i.e., capability, opportunity, and motivation). Moreover, our Cronbach alpha results suggest that the three sub-scales have acceptable internal consistency (α > 0.70). Although our measure included items aligned with the six COM-B constructs (i.e., physical capability, psychological capability, physical opportunity, social opportunity, reflective motivation, and automatic motivation), we opted for a more parsimonious three-factor solution. Previous studies have identified an inverse association between questionnaire length and response rate [53] and researchers often encounter difficulties in persuading teachers to complete follow-up surveys in school-based research. This is especially true in large-scale dissemination studies, which have lower response rates than feasibility, efficacy, and effectiveness trials [54–56].

Teachers play an important role in the delivery of school-based physical activity interventions, but few studies have examined the impact of implementation support strategies on teacher level determinants (e.g., feasibility, acceptability, and capability). Ryan and colleagues [25] found evidence to support the use of the behavior change techniques ‘Action Planning’ ‘and ‘Feedback on behavior’ in staff training to increase students’ physical activity. However, the authors noted a lack of thorough reporting on the implementation of school-based physical activity interventions and highlighted the need for valid and reliable tools [25]. As such, there is need for pragmatic measures that are feasible to use in real-world settings, such as schools [57]. The COM-PASS addresses this shortfall and may have utility for measuring the impact of implementation support strategies on teachers’ capability, opportunity, and motivation to deliver physical activity programs and policies in schools.

### Future research

As noted by Beets and colleagues [58] in their Theory of Expanded, Extended and Enhanced Opportunities for youth physical activity, teachers are largely responsible for the effects of school-based physical activity interventions by creating new opportunities for students to be active at school (expanding), making existing opportunities longer (extending), and making the most out of existing opportunities (enhancing). We encourage researchers to use the COM-PASS to explore the role of teachers’ competence, opportunity, and motivation, as mediators of the intervention effect on students’ physical activity levels. We also encourage researchers to conduct further validation studies of the COM-PASS in diverse samples of primary and secondary school teachers. For example, future studies should examine the test–retest reliability and responsiveness of the COM-PASS. There is also a need for further studies to examine the appropriateness of the tool when adapted for the evaluation of different physical activity programs and policies.

### Strengths and limitations

A notable strength of this study is the involvement of academic experts and teachers to develop a pragmatic tool. In addition, our measure was developed using the COM-B model, which has been identified as an appropriate framework for assessing and guiding physical activity interventions [17, 29]. However, there are some limitations that should be noted. First, most of the participants in Phase 3 (i.e., factorial validity) were Australian secondary school teachers. Further studies examining the factorial validity of the COM-PASS in primary and secondary teachers across the globe are needed. Second, the sample size involved in our factorial validity study was below the > 250 participant threshold recommended for confirmatory factors analyses [45]. It is important to note that our study was conducted during the post COVID-19 period, when schools and teachers were experiencing high levels of disruption and absenteeism [59]. Despite these limitations, our findings provide preliminary evidence for the content and structural validity of the COM-PASS.

## Conclusions

The development and evaluation of the COM-PASS tool represents an important step towards bridging the gap between research and practice in school-based physical activity research. Our research has shown that the COM-PASS has good content validity, internal consistency, and structural validity. We have also demonstrated that the measure is considered appropriate by teachers. We developed the COM-PASS to help researchers navigate the design, evaluation, and dissemination of school-based physical activity interventions. The tool may also have utility in university and school settings for evaluating the effects of physical activity courses for preservice and in-service teachers. The COM-PASS is free to use and is available upon request from the corresponding author.

### Supplementary Information

Supplementary Material 1: Supplementary file 1. Initial COM-PASS items (Phase 1: Delphi study, round 1). Supplementary file 2. Interview script and COM-PASS tested using the ‘think-aloud’ approach(Phase 2: Teacher interviews). Supplementary file 3. Correlation matrix (figures in parentheses are *P* values) of the COM-PASS items, M and SD. 

## Data Availability

The datasets used and/or analyzed during the current study are available from the corresponding author on reasonable request.

## Appendix 1

The final COM-PASS items

**Table:**

The following items refer to your confidence, opportunity, and motivation to deliver the [*insert physical activity policy or program name)* in your school. Please select one option per item to indicate how much you agree or disagree with each statement	Strongly disagree	Disagree	Neutral	Agree	Strongly agree
I have the physical fitness (e.g., aerobic and muscular fitness, flexibility) to deliver the [*insert physical activity program or policy name*]	SD	D	N	A	SA
I have the physical skills (e.g., I can demonstrate the activities) to deliver the [*insert physical activity program or policy name*]	SD	D	N	A	SA
I know how to deliver the [*insert physical activity program or policy name*]	SD	D	N	A	SA
I can deliver the [*insert physical activity program or policy name*] even when barriers emerge (e.g., lack of student engagement or lack of time)	SD	D	N	A	SA
My school has the physical facilities (e.g., access to a gym or appropriate indoor or outdoor space) to deliver the [*insert physical activity program or policy name*]	SD	D	N	A	SA
My school has the equipment (e.g., resistance bands, balls, activity cards) to deliver the [*insert physical activity program or policy name*]	SD	D	N	A	SA
I have enough time to plan the delivery of the [*insert physical activity program or policy name*]	SD	D	N	A	SA
I have the necessary support from school executives (e.g., principal or head of department) to deliver the [*insert physical activity program or policy name*]	SD	D	N	A	SA
I have the necessary support from my colleagues to deliver the [*insert physical activity program or policy name*]	SD	D	N	A	SA
I have the necessary support from parents and guardians to deliver the [*insert physical activity program or policy name*]	SD	D	N	A	SA
I can see the benefits (e.g., improvements in students’ classroom behavior) of delivering the [*insert physical activity program or policy name*]	SD	D	N	A	SA
I am motivated to deliver the [*insert physical activity program or policy name*]	SD	D	N	A	SA
I enjoy delivering the [*insert physical activity program or policy name*]	SD	D	N	A	SA
Delivering the [*insert physical activity program or policy name*] can become part of my school routine	SD	D	N	A	SA

*SD* Strongly disagree, *D* Disagree, *N* Neutral, *A* Agree, *‘SA* Strongly agree

## Abbreviations

CFA: Confirmatory Factor Analysis
CFI: Comparative Fit Index
COM-B: Capability, Opportunity, and Motivation behavior
COM-PASS: Capability, Opportunity, and Motivation to deliver Physical Activity in School Scale
RMSEA: Root Mean Square Error of Approximation
TLI: Tucker-Lewis Index

## References

[1] World Health Organisation. Global status report on physical activity 2022: country profiles. Geneva: World Health Organization; 2022.

[2] Aubert S, et al. Global matrix 4.0 physical activity report card grades for children and adolescents: results and analyses from 57 countries. J Phys Act Health. 2022;19(11):700–28. 36280233 10.1123/jpah.2022-0456

[3] Dumith SC, et al. Physical activity change during adolescence: a systematic review and a pooled analysis. Int J Epidemiol. 2011;40(3):685–98. 21245072 10.1093/ije/dyq272

[4] Farooq A, et al. Longitudinal changes in moderate-to-vigorous-intensity physical activity in children and adolescents: A systematic review and meta-analysis. Obes Rev. 2020;21(1): e12953. 31646739 10.1111/obr.12953 PMC6916562

[5] Martins J, et al. Adolescents’ perspectives on the barriers and facilitators of physical activity: an updated systematic review of qualitative studies. Int J Environ Res Public Health. 2021;18(9):4954. 34066596 10.3390/ijerph18094954 PMC8125166

[6] Martins J, et al. Adolescents’ perspectives on the barriers and facilitators of physical activity: a systematic review of qualitative studies. Health Educ Res. 2015;30(5):742–55. 26324394 10.1093/her/cyv042

[7] Mehtälä MAK, et al. A socio-ecological approach to physical activity interventions in childcare: a systematic review. Int J Behav Nutr Phys Act. 2014;11:1–12. 24559188 10.1186/1479-5868-11-22 PMC3936868

[8] Holman RM, Carson V, Janssen I. Does the fractionalization of daily physical activity (sporadic vs. bouts) impact cardiometabolic risk factors in children and youth? PloS One. 2011;6(10). 21998688 10.1371/journal.pone.0025733 PMC3187782

[9] Metcalf B, Henley W, Wilkin T. Effectiveness of intervention on physical activity of children: systematic review and meta-analysis of controlled trials with objectively measured outcomes (EarlyBird 54). BMJ. 2012;345:e5888. 23044984 10.1136/bmj.e5888

[10] Love R, Adams J, van Sluijs EM. Are school-based physical activity interventions effective and equitable? A meta-analysis of cluster randomized controlled trials with accelerometer-assessed activity. Obes Rev. 2019;20(6):859–70. 30628172 10.1111/obr.12823 PMC6563481

[11] Borde R, et al. Methodological considerations and impact of school-based interventions on objectively measured physical activity in adolescents: a systematic review and meta-analysis. Obes Rev. 2017;18(4):476–90. 28187241 10.1111/obr.12517

[12] Hartwig TB, et al. School-based interventions modestly increase physical activity and cardiorespiratory fitness but are least effective for youth who need them most: an individual participant pooled analysis of 20 controlled trials. Br J Sports Med. 2021;55(13):721–9. 10.1136/bjsports-2020-102740 33441332

[13] Neil-Sztramko SE, Caldwell H, Dobbins M. School-based physical activity programs for promoting physical activity and fitness in children and adolescents aged 6 to 18. Cochrane Database Syst Rev. 2021;9:CD007651. 34555181 10.1002/14651858.CD007651.pub3 PMC8459921

[14] Jago R, et al. Rethinking children’s physical activity interventions at school: A new context-specific approach. Front Public Health. 2023;11:1272. 10.3389/fpubh.2023.1149883 PMC10133698 37124783

[15] Porter A, et al. Physical activity interventions in European primary schools: a scoping review to create a framework for the design of tailored interventions in European countries. Front Public Health. 2024;12:1321167. 38389941 10.3389/fpubh.2024.1321167 PMC10883314

[16] Barnes C, et al. Improving implementation of school-based healthy eating and physical activity policies, practices, and programs: a systematic review. Trans Behav Med. 2021;11(7):1365–410. 34080618 10.1093/tbm/ibab037 PMC8320878

[17] Rosenkranz RR, et al. Physical activity capability, opportunity, motivation and behavior in youth settings: theoretical framework to guide physical activity leader interventions. Int Rev Sport Exerc Psychol. 2023;16(1):529–53. 10.1080/1750984X.2021.1904434

[18] Hartikainen J, et al. Classroom-based physical activity and teachers’ instructions on students’ movement in conventional classrooms and open learning spaces. Learning Environ Res. 2023;26(1):177–98. 10.1007/s10984-022-09411-3

[19] Mak TC, Chan DK, Capio CM. Strategies for teachers to promote physical activity in early childhood education settings—a scoping review. Int J Environ Res Public Health. 2021;18(3):867. 33498374 10.3390/ijerph18030867 PMC7908495

[20] Naylor P-J, et al. Implementation of school based physical activity interventions: a systematic review. Prev Med. 2015;72:95–115. 25575800 10.1016/j.ypmed.2014.12.034

[21] Cox A, Noonan RJ, Fairclough SJ. PE teachers’ perceived expertise and professional development requirements in the delivery of muscular fitness activity: PE Teacher EmPOWERment Survey. Eur Phys Educ Rev. 2023;29(2):251–67.

[22] Kennedy SG, et al. Evaluating the reach, effectiveness, adoption, implementation and maintenance of the Resistance Training for Teens program. Int J Behav Nutr Phys Act. 2021;18:1–18. 34496861 10.1186/s12966-021-01195-8 PMC8425054

[23] Wolfenden L, et al. Strategies for enhancing the implementation of school‐based policies or practices targeting diet, physical activity, obesity, tobacco or alcohol use. Cochrane Database Syst Rev. 2022;8(8):CD011677. 10.1002/14651858.CD011677.pub3 PMC9422950 36036664

[24] Lander N, et al. Characteristics of teacher training in school-based physical education interventions to improve fundamental movement skills and/or physical activity: A systematic review. Sports Med. 2017;47:135–61. 27294354 10.1007/s40279-016-0561-6

[25] Ryan M, et al. Features of effective staff training programmes within school-based interventions targeting student activity behaviour: a systematic review and meta-analysis. Int J Behav Nutr Phys Act. 2022;19(1):1–23. 36153617 10.1186/s12966-022-01361-6 PMC9509574

[26] Nathan N, et al. Barriers and facilitators to the implementation of physical activity policies in schools: a systematic review. Prev Med. 2018;107:45–53. 29155228 10.1016/j.ypmed.2017.11.012

[27] Nilsen P. Making sense of implementation theories, models, and frameworks. Implement Sci. 2015;10:53. 25895742 10.1186/s13012-015-0242-0 PMC4406164

[28] McKay H, et al. Implementation and scale-up of physical activity and behavioural nutrition interventions: an evaluation roadmap. Int J Behav Nutr Phys Act. 2019;16(1):102. 31699095 10.1186/s12966-019-0868-4 PMC6839114

[29] Michie S, Atkins L, West R. The behaviour change wheel. In: A guide to designing interventions. 1st ed. Great Britain: Silverback Publishing; 2014. p. 1003–1010.

[30] Damschroder LJ, et al. The updated Consolidated Framework for Implementation Research based on user feedback. Implement Sci. 2022;17(1):75. 36309746 10.1186/s13012-022-01245-0 PMC9617234

[31] Mokkink LB, et al. The COSMIN checklist for assessing the methodological quality of studies on measurement properties of health status measurement instruments: an international Delphi study. Qual Life Res. 2010;19:539–49. 20169472 10.1007/s11136-010-9606-8 PMC2852520

[32] QuestionPro. QuestionPro Survey Software. 2024.

[33] Keyworth C, et al. Acceptability, reliability, and validity of a brief measure of capabilities, opportunities, and motivations (“COM-B”). Br J Health Psychol. 2020;25(3):474–501. 32314500 10.1111/bjhp.12417

[34] Powell C. The Delphi technique: myths and realities. J Adv Nurs. 2003;41(4):376–82. 12581103 10.1046/j.1365-2648.2003.02537.x

[35] Messick S. Standards of validity and the validity of standards in performance asessment. Educ Meas Issues Pract. 1995;14(4):5–8. 10.1111/j.1745-3992.1995.tb00881.x

[36] Vogt DS, King DW, King LA. Focus groups in psychological assessment: enhancing content validity by consulting members of the target population. Psychol Assess. 2004;16(3):231. 15456379 10.1037/1040-3590.16.3.231

[37] Oremus M, Cosby JL, Wolfson C. A hybrid qualitative method for pretesting questionnaires: the example of a questionnaire to caregivers of Alzheimer disease patients. Res Nurs Health. 2005;28(5):419–30. 16163677 10.1002/nur.20095

[38] Willis GB. Cognitive interviewing: a tool for improving questionnaire design. Thousand Oaks, CA: Sage publications; 2004.

[39] Sylvester BD, et al. Perceived variety, psychological needs satisfaction and exercise-related well-being. Psychol Health. 2014;29(9):1044–61. 24669787 10.1080/08870446.2014.907900

[40] Strauss A, Corbin J. Basics of qualitative research techniques. Thousand Oaks, CA: Sage; 1998.

[41] Linn RL. The standards for educational and psychological testing: Guidance in test development. In Downing SM, Haladyna TM (Eds.), Handbook of test development. Mahwah, NJ: Erlbaum; 2006. p. 27–38.

[42] Thomas Kelly H, et al. Supporting adolescents’ participation in muscle-strengthening physical activity: protocol for the ‘Resistance Training for Teens’(RT4T) hybrid type III implementation–effectiveness trial. BMJ Open. 2023;13(11):e075488. 10.1136/bmjopen-2023-075488 PMC10626834 37914300

[43] Bandalos DL, Finney SJ. Factor analysis: Exploratory and confirmatory. In: The reviewer’s guide to quantitative methods in the social sciences. New York, NY: Routledge; 2018. p. 98–122.

[44] Arbuckle J. Amos (Version 26.0)[Computer Program]. Chicago: IBM SPSS; 2019.

[45] Hu LT, Bentler PM. Cutoff criteria for fit indexes in covariance structure analysis: Conventional criteria versus new alternatives. Struct Equ Modeling. 1999;6(1):1–55. 10.1080/10705519909540118

[46] Tucker LR, Lewis C. A reliability coefficient for maximum likelihood factor analysis. Psychometrika. 1973;38(1):1–10. 10.1007/BF02291170

[47] Browne MW, Cudeck R. Alternative ways of assessing model fit. Sociol Methods Res. 1992;21(2):230–58. 10.1177/0049124192021002005

[48] Xia Y, Yang Y. RMSEA, CFI, and TLI in structural equation modeling with ordered categorical data: The story they tell depends on the estimation methods. Behav Res Methods. 2019;51:409–28. 29869222 10.3758/s13428-018-1055-2

[49] Marsh HW, et al. Factorial, convergent, and discriminant validity of timss math and science motivation measures: A comparison of Arab and Anglo-Saxon countries. J Educ Psychol. 2013;105(1):108. 10.1037/a0029907

[50] Downey RG, King CV. Missing data in Likert ratings: A comparison of replacement methods. J Gen Psychol. 1998;125(2):175–91. 9935342 10.1080/00221309809595542

[51] DuBay WH. The principles of readability. Online Submission. 2004.

[52] Meyer GS, et al. More quality measures versus measuring what matters: a call for balance and parsimony. BMJ Qual Saf. 2012;21(11):964–8. 22893696 10.1136/bmjqs-2012-001081 PMC3594932

[53] Rolstad S, Adler J, Rydén A. Response burden and questionnaire length: is shorter better? A review and meta-analysis. Value in Health. 2011;14(8):1101–8. 22152180 10.1016/j.jval.2011.06.003

[54] Riley N, et al. Dissemination of thinking while moving in maths: Implementation barriers and facilitators. Transl J Am Coll Sports Med. 2021;6(1):e000148.

[55] Kennedy SG, et al. Implementation at-scale of school-based physical activity interventions: A systematic review utilizing the RE-AIM framework. Obes Rev. 2021;22: e13184. 33527738 10.1111/obr.13184

[56] Kennedy SG, et al. Evaluating the reach, effectiveness, adoption, implementation and maintenance of the Resistance Training for Teens program. Int J Behav Nutr Phys Act. 2021;18:122. 34496861 10.1186/s12966-021-01195-8 PMC8425054

[57] Glasgow RE, Riley WT. Pragmatic measures: what they are and why we need them. Am J Prev Med. 2013;45(2):237–43. 23867032 10.1016/j.amepre.2013.03.010

[58] Beets M, et al. The theory of expanded, extended, and enhanced opportunities for youth physical activity promotion. Int J Behav Nutr Phys Act. 2016;13(1):120. 27852272 10.1186/s12966-016-0442-2 PMC5112641

[59] Gore J, et al. The impact of COVID-19 on student learning in New South Wales primary schools: an empirical study. Aust Educ Res. 2021;48:605–37. 10.1007/s13384-021-00436-w PMC7952260 33727761

