# Supplementary material for: Development and evaluation of the Capability, Opportunity, and Motivation to deliver Physical Activity in School Scale (COM-PASS)
Source: Int J Behav Nutr Phys Act. 2024 Aug 26;21:93. doi: 10.1186/s12966-024-01640-4 (PMC11346190; doi:10.1186/s12966-024-01640-4)
Supplement: Supplementary file 1 — Supplementary Material 1: Supplementary file 1. Initial COM-PASS items (Phase 1: Delphi study, round 1). Supplementary file 2. Interview script and COM-PASS tested using the ‘think-aloud’ approach(Phase 2: Teacher interviews). Supplementary file 3. Correlation matrix (figures in parentheses are P values) of the COM-PASS items, M and SD. [file 12966_2024_1640_MOESM1_ESM.docx]

**Supplementary material 1:** **Initial COM-PASS items (Phase 1: Delphi study, round 1)**

| **5-point scale ranging from ’1 = *Poor match*’ to ’5 = *Excellent match*’** | | | | | | |
| --- | --- | --- | --- | --- | --- | --- |
|  | | 1 | 2 | 3 | 4 | 5 |
| **Physical capability**  Definition^1^: 'Physical skill, strength or stamina' | | | | | | |
| 1. Indicate the degree to which Q1 and Q2 matches the definition of physical capability: | | | | | | |
| **Q1.** I have the physical fitness to deliver the [*physical activity program or policy*] | | **❏** | **❏** | **❏** | **❏** | **❏** |
| **Q2.** I have physical skills to deliver the [*physical activity program or policy*] | | **❏** | **❏** | **❏** | **❏** | **❏** |
| Suggestions for amendments (optional): | |  | | | | |
| **Psychological capability**  Definition^1^: 'Knowledge or psychological skills, strength, or stamina to engage in the necessary mental processes' | | | | | | |
| 2. Indicate the degree to which Q3 and Q4 matches the definition of psychological capability: | | | | | | |
| **Q3.** I have the knowledge to deliver the [*physical activity program or policy*] | | **❏** | **❏** | **❏** | **❏** | **❏** |
| **Q4.** I have the confidence to deliver the [*physical activity program or policy*] | | **❏** | **❏** | **❏** | **❏** | **❏** |
| Suggestions for amendments (optional): | |  | | | | |
| **Physical opportunity**  Definition^1^: 'Opportunity afforded by the environment involving time, resources, locations, cues, physical 'affordance'' | | | | | | |
| 3. Indicate the degree to which Q5-Q7 matches the definition of physical opportunity: | | | | | | |
| **Q5.** My school has the physical facilities (e.g., access to a gym or appropriate space) to deliver the [*physical activity program or policy*] | | **❏** | **❏** | **❏** | **❏** | **❏** |
| **Q6.** My school has the equipment (e.g., resistance bands) to deliver the [*physical activity program or policy*] | | **❏** | **❏** | **❏** | **❏** | **❏** |
| **Q7.** I have the time to prepare to deliver the [*physical activity program or policy*] | | **❏** | **❏** | **❏** | **❏** | **❏** |
| Suggestions for amendments (optional): | |  | | | | |
| **Social opportunity**  Definition^1^: 'Opportunity afforded by interpersonal influences, social cues and cultural norms that influence the way that we think about things, e.g., the words and concepts that make up our language' | | | | | | |
| 4. Indicate the degree to which Q8 and Q9 matches the definition of social opportunity: | | | | | | |
| **Q8.** I have the necessary support from school executives (e.g., principal) to deliver the [*physical activity program or policy*] | | **❏** | **❏** | **❏** | **❏** | **❏** |
| **Q9.** I have the necessary departmental support (e.g., Head of Department) to deliver the [*physical activity program or policy*] | | **❏** | **❏** | **❏** | **❏** | **❏** |
| Suggestions for amendments (optional): | |  | | | | |
| **Reflective motivation**  Definition^1^: 'Reflective processes involving plans (self-conscious intentions) and evaluations (beliefs about what is good and bad)' | | | | | | |
| 5. Indicate the degree to which Q10 matches the definition of reflective motivation: | | | | | | |
| **Q10.** I can see the benefits of delivering the [*physical activity program or policy*] | | **❏** | **❏** | **❏** | **❏** | **❏** |
| Suggestions for amendments (optional): | |  | | | | |
| **Automatic motivation**  Definition^1^: Automatic processes involving emotional reactions, desires (wants and needs), impulses, inhibitions, drive states and reflex responses' | | | | | | |
| 6. Indicate the degree to which Q11-Q13 matches the definition of automatic motivation: | | | | | | |
| **Q11.** I am motivated to deliver the [*physical activity program or policy*] | | **❏** | **❏** | **❏** | **❏** | **❏** |
| **Q12.** Other teachers in my department are motivated to deliver the [*physical activity program or policy*] | | **❏** | **❏** | **❏** | **❏** | **❏** |
| **Q13.** My students are motivated to participate in the [*physical activity program or policy*] | | **❏** | **❏** | **❏** | **❏** | **❏** |
| Suggestions for amendments (optional): | |  | | | | |
| 7. What answer format for these COM-B items would you suggest (*select one*)? | | | | | | |
| **❏** 10-point Liker scale with ‘1 = Strongly disagree’ to ’10 = Strongly agree’ (see image)  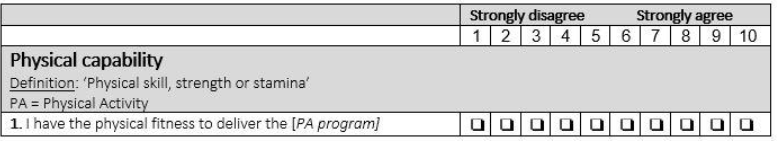 | | | | | | |
| **❏** 5-point Likert scale with ‘’1 = Strongly disagree (SD), 2 = Disagree (D), 3 = Neutral (N), 4 = Agree (A) and 5 = Strongly agree (SA)’’ (see image)  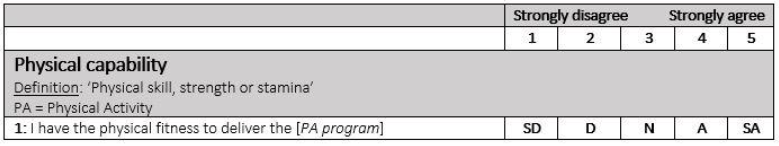 | | | | | | |
| 8. Any other comments? |  | | | | | |

^1^ Michie, S., Atkins, L., & West, R. (2014). The behaviour change wheel. *A guide to designing interventions. 1st ed. Great Britain: Silverback Publishing*, 1003-1010.

**Supplementary material 2: Interview script and COM-PASS tested using the ‘think-aloud’ approach (Phase 2: Teacher interviews)**

*[script for interviewer (A.V.)]*

Dear Teacher,

Thank you for participating in this study.

We are developing a survey to assess teachers’ capability, opportunity, and motivation to deliver primary and secondary school-based physical activity programs. Since this survey will be for teachers, it is important that all questions make sense to teachers. Your feedback on our survey is therefore of great value.

It will take about 20 minutes. Before we start, could you please let me know if you give your consent to your contribution to the development to this teacher questionnaire and that I am going to record this for research purposes? (*A.V. reads and ticks the two boxes below if teacher says yes – if no, the interview will be ended*):

❏ I give **consent** to my contribution to the development of this teacher questionnaire and receiving a 20-dollar voucher as a thank for my time.

❏ I agree with this interview being **recorded** for research purposes and understand my responses will be processed anonymously.

| **Background information** |
| --- |
| Name: |
| Email address: |
| Current role: 1. Primary school teacher 2. Secondary school teacher (*please circle)* |

The survey exists of 17 questions. What I want you to do is read each question aloud, one a at time, then consider the following: ‘*’What, in your own words, does this question mean to me?*’’.

*[A.V. reads the example below]*

| **Physical activity program description** |
| --- |
| 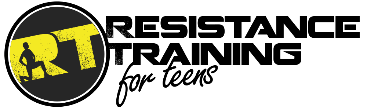**Resistance Training for Teens** Resistance Training for Teens is an evidence-based 8-week program for year 9 and 10 students including 8 sessions (practical and theoretical). The program is designed to provide adolescents with competence, confidence, and motivation (i.e., physical literacy) to engage in muscle-strengthening activities. |

This is just an example of a program so you know what kind of programs teachers could think of when answering the questions. As you can see, the questions have some definitions as well and it would be great if you can also read the definitions aloud before reading the questions.

Do you have any questions before we start? Okay, let’s start with reading the first definition followed by the questions and telling me what you think it means to you, in your own words!

|  | Strongly  Disagree | Disagree | Neutral | Agree | Strongly Agree |
| --- | --- | --- | --- | --- | --- |
| **Physical capability**  Definition: ‘Physical skill, strength or stamina’ | | | | | |
| 1. I have the physical fitness (i.e., flexibility, aerobic and muscular fitness) to deliver the [*physical activity program or policy*] | SD | D | N | A | SA |
| 2. I have the physical skills (e.g., I can demonstrate the activities) to deliver the [*physical activity program or policy*] | SD | D | N | A | SA |
| **Psychological capability**  Definition: ‘Knowledge or psychological skills, strength, or stamina to engage in the necessary mental processes’ | | | | | |
| 3. I know how to deliver the [*physical activity program or policy*] | SD | D | N | A | SA |
| 4. I can deliver the [*physical activity program or policy*] even when barriers emerge (e.g., lack of time) | SD | D | N | A | SA |
| **Physical opportunity**  Definition: ‘Opportunity afforded by the environment involving time, resources, locations, cues, physical ‘affordance’’ | | | | | |
| 5. My school has the physical facilities (e.g., access to a gym or appropriate indoor or outdoor space) to deliver the [*physical activity program or policy*] | SD | D | N | A | SA |
| 6. My school has the equipment (e.g., resistance bands, balls, activity cards) to deliver the [*physical activity program or policy*] | SD | D | N | A | SA |
| 7. I have enough time to plan the delivery of the [*physical activity program or policy*] | SD | D | N | A | SA |
| 8. I have enough time to deliver the [*physical activity program or policy*] | SD | D | N | A | SA |
| **Social opportunity**  Definition: ‘Opportunity afforded by interpersonal influences, social cues and cultural norms that influence the way that we think about things, e.g., the words and concepts that make up our language’ | | | | | |
| 9. I have the necessary support from school executives (e.g., principal or Head of Department) to deliver the [*physical activity program or policy*] | SD | D | N | A | SA |
| 10. I have the necessary support from my colleagues to deliver the [*physical activity program or policy*] | SD | D | N | A | SA |
| 11. I have the necessary support from parents and guardians to deliver the [*physical activity program or policy*] | SD | D | N | A | SA |
| **Reflective motivation**  Definition: ‘Reflective processes involving plans (self-conscious intentions) and evaluations (beliefs about what is good and bad)’ | | | | | |
| 12. I can see the benefits (e.g., improvements in students’ classroom behaviour) of delivering the [*physical activity program or policy*] | SD | D | N | A | SA |
| 13. I am planning to deliver the [*physical activity program or policy*] | SD | D | N | A | SA |
| 14. I am motivated to deliver the [*physical activity program or policy*] | SD | D | N | A | SA |
| 15. My students are motivated to participate in the [*physical activity program or policy*] | SD | D | N | A | SA |
| **Automatic motivation**  Definition: ‘Automatic processes involving emotional reactions, desires (wants and needs), impulses, inhibitions, drive states and reflex responses’ | | | | | |
| 16. I enjoy delivering the [*physical activity program or policy*] | SD | D | N | A | SA |
| 17. Delivering the [*physical activity program or policy*] is part of my routine | SD | D | N | A | SA |

We are almost done! Only a few final questions:

- Did the answer choices include your answer?
- Did you understand how to answer the questions?
- Did the questionnaire leave anything out you felt was important?

This was it! Do you have any final comments?

Many thanks again and I will send you the voucher via email.

*[end of interview]*

**Supplementary material 3: Correlation matrix of COM-PASS items**

| Items | PHC1 | PHC2 | PSC1 | PSC2 | PHO1 | PHO2 | PHO3 | SO1 | SO2 | SO3 | RM1 | RM2 | AM1 | AM2 |
| --- | --- | --- | --- | --- | --- | --- | --- | --- | --- | --- | --- | --- | --- | --- |
| PHC1 | 1.000 |  |  |  |  |  |  |  |  |  |  |  |  |  |
| PHC2 | .818 (.000) | 1.000 |  |  |  |  |  |  |  |  |  |  |  |  |
| PSC1 | .378  (.000) | .387  (.000) | 1.000 |  |  |  |  |  |  |  |  |  |  |  |
| PSC2 | .271  (.000) | .301  (.000) | .450  (.000) | 1.000 |  |  |  |  |  |  |  |  |  |  |
| PHO1 | .314  (.000) | .265  (.000) | .359  (.000) | .350  (.000) | 1.000 |  |  |  |  |  |  |  |  |  |
| PHO2 | .080  (.131) | .117  (.051) | .326  (.000) | .208  (.002) | .431  (.000) | 1.000 |  |  |  |  |  |  |  |  |
| PHO3 | .065  (.182) | .080  (.134) | .201  (.002) | .311  (.000) | .232  (.001) | .262  (.000) | 1.000 |  |  |  |  |  |  |  |
| SO1 | .148  (.019) | .117  (.051) | .125  (.041) | .150  (.018) | .230  (.001) | .250  (.000) | .478  (.000) | 1.000 |  |  |  |  |  |  |
| SO2 | .262  (.000) | .234  (.000) | .254  (.000) | .309  (.000) | .355  (.000) | .343  (.000) | .369  (.000) | .468  (.000) | 1.000 |  |  |  |  |  |
| SO3 | .178  (.006) | .203  (.002) | .264  (.000) | .327  (.000) | .400  (.000) | .290  (.000) | .428  (.000) | .358  (.000) | .502  (.000) | 1.000 |  |  |  |  |
| RM1 | .233  (.001) | .182  (.005) | .288  (.000) | .244  (.000) | .328  (.000) | .128  (.037) | .160  (.013) | .104  (.073) | .241  (.000) | .346  (.000) | 1.000 |  |  |  |
| RM2 | .262  (.000) | .256  (.000) | .265  (.000) | .256  (.000) | .345  (.000) | .263  (.000) | .340  (.000) | .379  (.000) | .445  (.000) | .409  (.000) | .398  (.000) | 1.000 |  |  |
| AM1 | .395  (.000) | .367  (.000) | .386  (.000) | .317  (.000) | .338  (.000) | .140  (.025) | .228  (.001) | .215  (.001) | .281  (.000) | .439  (.000) | .406  (.000) | .611  (.000) | 1.000 |  |
| AM2 | .315  (.000) | .288  (.000) | .287  (.000) | .308  (.000) | .272  (.000) | .212  (.001) | .446  (.000) | .305  (.000) | .387  (.000) | .537  (.000) | .436  (.000) | .603  (.000) | .656  (.000) | 1.000 |
| *M* | 4.34 | 4.32 | 3.21 | 3.45 | 3.99 | 2.99 | 3.13 | 3.72 | 3.88 | 3.70 | 4.30 | 4.12 | 4.04 | 3.88 |
| *SD* | .860 | .813 | 1.009 | .806 | .936 | 1.361 | .924 | .826 | .801 | .775 | .774 | .748 | .729 | .761 |
| Minimum | 1 | 1 | 1 | 1 | 1 | 1 | 1 | 1 | 1 | 1 | 1 | 1 | 1 | 1 |
| Maximum | 5 | 5 | 5 | 5 | 5 | 5 | 5 | 5 | 5 | 5 | 5 | 5 | 5 | 5 |
| Sample size | 196 | 196 | 196 | 196 | 196 | 196 | 196 | 196 | 196 | 196 | 196 | 196 | 196 | 196 |
